# A Contactless Method for Measuring Full-Day, Naturalistic Motor Behavior Using Wearable Inertial Sensors

**DOI:** 10.3389/fpsyg.2021.701343

**Published:** 2021-10-22

**Authors:** John M. Franchak, Vanessa Scott, Chuan Luo

**Affiliations:** Perception, Action, and Development Laboratory, Department of Psychology, University of California, Riverside, Riverside, CA, United States

**Keywords:** motor development, posture, body position, wearable sensors, human activity recognition, machine learning

## Abstract

How can researchers best measure infants' motor experiences in the home? Body position—whether infants are held, supine, prone, sitting, or upright—is an important developmental experience. However, the standard way of measuring infant body position, video recording by an experimenter in the home, can only capture short instances, may bias measurements, and conflicts with physical distancing guidelines resulting from the COVID-19 pandemic. Here, we introduce and validate an alternative method that uses machine learning algorithms to classify infants' body position from a set of wearable inertial sensors. A laboratory study of 15 infants demonstrated that the method was sufficiently accurate to measure individual differences in the time that infants spent in each body position. Two case studies showed the feasibility of applying this method to testing infants in the home using a contactless equipment drop-off procedure.

## 1. Introduction

Infants' increasing ability to transition into and maintain balance in different body positions is a hallmark of the first year (Adolph and Franchak, 2017). At birth, newborns can only lay supine on their backs or prone on their bellies. Otherwise, they rely on caregivers to place them in different positions or hold them in their arms. With age, infants master the ability to sit independently, crawl in a prone position, stand upright, and walk. In this paper, we describe a new method to characterize infants' body positions—held by caregivers, supine, prone, sitting, and upright—across an entire day using machine learning classification of wearable inertial motion sensors. We begin by describing the importance of understanding infant body position and then review existing measurement approaches. Afterwards, we present two studies: A laboratory validation study that shows how wearable sensors can be used to accurately categorize infant body position, and case studies that demonstrate how feasibly the method can be adapted to collect data from infants in the home while relying on caregivers to administer the procedure.

Growing evidence suggests that acquiring more advanced control over body position augments infants' opportunities for learning and exploration (Gibson, 1988; Libertus and Hauf, 2017; Franchak, 2020). For example, infants' visual experiences differ according to body position: While prone, infants' field of view is dominated by the ground surface and objects near the body, whereas upright infants have a more expansive view of their surroundings that includes distant objects and faces (Franchak et al., 2011, 2018; Kretch et al., 2014; Luo and Franchak, 2020). Sitting facilitates visual and manual exploration of objects compared with laying prone or supine (Soska and Adolph, 2014; Luo and Franchak, 2020). Upright locomotion (walking) compared with prone locomotion (crawling) allows infants to travel farther, more easily carry objects, and elicits different social responses from caregivers (Gibson, 1988; Adolph and Tamis-LeMonda, 2014; Karasik et al., 2014). Accordingly, learning to sit and walk is linked with downstream improvements in language learning and spatial cognition (Soska et al., 2010; Oudgenoeg-Paz et al., 2012, 2015; Walle and Campos, 2014; He et al., 2015; Walle, 2016; West et al., 2019, c.f. Moore et al., 2019). Presumably, these facilitative effects result from infants spending more time sitting, standing, and walking. For example, mastering the ability to sit independently nearly doubled the amount of time that 6-month-olds spent sitting (both independent and supported sitting) in daily life compared with 6-month-old non-sitters (Franchak, 2019). Infants who spend more time sitting have increased opportunities to explore objects. Yet, little data are available to describe how infants spend their time in different body positions across a typical day, and how the prevalence of different body positions changes with age and motor ability.

Video observation is the gold standard for measuring body position. But, video observation comes with several costs, especially with respect to the goal of describing natural, home experiences across a full day. Whereas, language researchers have profitably used day-long audio recordings to characterize the everyday language experiences of infants (e.g., Weisleder and Fernald, 2013; Bergelson et al., 2019), motor researchers have been limited to scoring body position recorded in relatively short (15–60 min) video observations (Karasik et al., 2011, 2015; Nickel et al., 2013; Thurman and Corbetta, 2017; Franchak et al., 2018). Although infants can wear an audio recorder that travels wherever they go, capturing infants' movements requires an experimenter to follow the infant from place to place while operating a camcorder. Furthermore, the presence of the experimenter in the home may lead to reactivity—altering infants' and caregivers' behaviors when observed (Tamis-LeMonda et al., 2017; Bergelson et al., 2019)—which threatens generalizability. Another threat to external validity is how time is sampled: A short visit from an experimenter scheduled at a convenient time is unlikely to be representative of the full spectrum of daily activities (e.g., nap routines, meal times, play, and errands) that may moderate motor behavior (Fausey et al., 2015; de Barbaro and Fausey, 2021; Kadooka et al., 2021). Other limitations of video observation are practical rather than scientific. Video recording an infant for an entire hour is laborious; to do so for an entire day would not be feasible. Even if it were possible to capture full day video recordings of an infant, frame-by-frame coding of body position would be a gargantuan task—slow but feasible in a small sample, but intractable at a larger scale—and storage of large, full-day video files creates a nontrivial data management challenge. As with audio, collecting video data in the home across an entire day presents challenges for maintaining participant privacy (Cychosz et al., 2020). Finally, physical distancing guidelines during the COVID-19 pandemic mean that an experimenter may not be permitted in the home to operate a video camera.

One alternative is to employ survey methods in lieu of direct observation. Surveys can be conducted remotely without an experimenter present in the home, addressing some limitations of video observation (i.e., reactivity, privacy, labor, data storage). Although retrospective diaries have been used to estimate infant body position and motor activity (Majnemer and Barr, 2005; Hnatiuk et al., 2013), their accuracy and reliability are questionable. For example, Majnemer and Barr (2005) asked caregivers to fill out a diary every 2–3 h to indicate the infants' position for each 5-min interval since the last entry. However, by 12 months of age infants change position an average of 2–4 times per minute when playing (Nickel et al., 2013; Thurman and Corbetta, 2017). Thus, it seems unlikely that a caregiver could accurately estimate the time spent in body positions using a retrospective diary. Ecological momentary assessment (EMA) is one alternative: Sending text message surveys to ask caregivers to report on infants' instantaneous body position every 1–2 h across the day provides a sparse, but accurate report (Franchak, 2019; Kadooka et al., 2021). Although this method may better capture full-day experiences compared with short video observation (and more accurately compared with retrospective diaries), it lacks the real-time position data that are provided by video coding.

Classifying body position from wearable sensors provides a third option that addresses the limitations of both video and survey methods. Lightweight inertial movement units (IMUs)—small sensors that contain an accelerometer and gyroscope—can be worn for the entire day or multiple days taped to the skin, embedded in clothing, or worn on a wristwatch (Cliff et al., 2009; de Barbaro, 2019; Lobo et al., 2019; Bruijns et al., 2020). Notably, an experimenter does need not to be present, and data can be recorded at a dense sampling rate in real time. Although video data must be collected and coded to train the classifier, the video-recorded portion can be brief (addressing privacy, data storage, and data coding labor concerns) while still providing a full-day measure of activity. Previous validation studies show that wearing lightweight sensors does not alter movements even in young infants (Jiang et al., 2018). Child and adult studies have successfully used wearable motion sensors to characterize the intensity of physical activity (e.g., sedentary vs. moderate-to-vigorous) using either cut points that set thresholds for different activity levels (Trost et al., 2012; Kuzik et al., 2015; Hager et al., 2017; Armstrong et al., 2019) or by training machine-learning algorithms to classify activity into different levels (Hagenbuchner et al., 2015; Trost et al., 2018).

Body position may be a more challenging behavior to classify compared with physical activity intensity. For example, an infant can be stationary or moving quickly while upright, suggesting that simple cut points or thresholds may not be suitable (Kwon et al., 2019). However, results from previous studies using machine learning to classify activity type in adults (Preece et al., 2009; Arif and Kattan, 2015) and children (Nam and Park, 2013; Zhao et al., 2013; Ren et al., 2016; Stewart et al., 2018) are encouraging. For example, Nam and Park (2013) used a support vector machine classifier to distinguish 11 activity types—including rolling, standing still, walking, crawling, and climbing—in a laboratory study of 16- to 29-month-olds. The classification accuracy was high (98.4%), suggesting that machine learning classification of wearable sensors may be sufficiently sensitive to differentiate the activities of young children.

Despite an abundance of work with children and adults, only a handful of studies have investigated infants. A number of studies have used sensors worn on the wrists or ankles to estimate the frequency of limb movements in typical and atypical development (Smith et al., 2017; Jiang et al., 2018). Hewitt et al. (2019) used commercially-available sensors to detect one type of body position, prone, to estimate caregivers' adherence to “Tummy Time” recommendations. Greenspan et al. (2021) estimated body position angle using pitch angle cut-points from a single sensor embedded in a garment in 3-month-olds. Yao et al. (2019) used a pair of sensors, one worn by the infant and one worn by the caregiver, to train machine learning models that were able to accurately classify the time infants spent held by caregivers. Notably, the Yao et al. study validated their method “in the wild” by collecting data in the home rather than relying only on a laboratory sample, which suggests the feasibility of this method for our proposed application. Finally, one previous study measured body position in 7-month-old infants using a set of 4 IMUs embedded in a garment (Airaksinen et al., 2020). With all 4 sensors (accuracy declined using a single sensor or a pair of sensors), researchers were able to distinguish between supine, side-lying, and prone positions with 98% accuracy using a machine learning model.

Although recent work provides an encouraging outlook for measuring body position in infants (Yao et al., 2019; Airaksinen et al., 2020; Greenspan et al., 2021), there are several open questions. First, because past studies of body position (Airaksinen et al., 2020; Greenspan et al., 2021) did not include caregivers holding infants as a category, it is unknown whether our proposed body position categories—prone, supine, sitting, upright, and held by caregiver—can be accurately classified. Held by caregivers is critical because infants' bodies may seem to be configured in a similar way to another position while held (e.g., a caregiver cradling an infant might be in a similar body position to when they are supine in a crib or on the floor). For this reason, angle cut-points like those used in past work (Greenspan et al., 2021) are unlikely to capture differences in the five positions we aim to classify. Unless we can accurately distinguish when infants are held, it would not be possible to account for their body position across the day because infants are held as much as 50% of the time in a typical day (as measured using EMA, Franchak, 2019). Although the Yao et al. study measured caregiver holding time (but not other body positions), they used a pair of sensors (one worn by the infant and one worn by the caregiver). It is unclear whether sensors worn only by infants would be able to detect when they are held. Second, the Airaksinen et al. study's categories included sitting, however, sitting in daily life can take many forms—sitting on a caregiver's lap, sitting in a restrained seat, or sitting independently on the floor—that may make it harder to detect in the wild. In the current study, we trained and tested sitting in a variety of forms to be sure that we can capture the variability we expect to find across a full day in the home. Third, although a benefit of classifying behavior from wearable sensors is that an experimenter does not need to be present for the entire day, the classifiers still need to be trained on a set of manually-coded ground truth data (e.g., body positions coded from video synchronized with sensor data). Given the regulatory issues arising from the COVID-19 pandemic, such as physical distancing and sanitation, we investigated the feasibility of using a stationary camera and sensors dropped off at participants' doorstep for training and validating a classifier without the researcher entering the home. But, it remains an open question whether an experimenter can remotely guide caregivers through the complex procedure of applying the sensors, synchronizing the sensors to the camera, and eliciting different body positions in view of the camera.

A remote drop-off procedure would have utility aside from addressing the immediate concerns of the COVID-19 pandemic. For families who feel uncomfortable with an experimenter visiting their homes, a remote drop-off provides a way to collect observational data without an experimenter's presence. Removing the need for an experimenter to spend an hour in the home—simply to pan a video camera—also reduces the experimenter's labor for collecting data. Most importantly, removing the experimenter's presence from the home—and the need to record video for long periods of time—can reduce reactivity. Indeed, caregivers spoke more to infants when video-recorded by a stationary camera than during an audio-only recording (Bergelson et al., 2019). Although our method uses a stationary camera, it is only needed for a brief video-recorded period followed by a full-day motion measurement (without video or experimenter presence). This will allow unobtrusive capture of behavior across a sufficiently long period to examine within-day variability of behavior (de Barbaro and Fausey, 2021) with minimum reactivity. Such data are crucial for testing the links between everyday experiences and subsequent development (Franchak, 2020). For example, one potential mechanism to explain why the acquisition of independent walking predicts increases in vocabulary development (Walle and Campos, 2014; Oudgenoeg-Paz et al., 2015) is that caregivers provide different language input to infants when infants are crawling compared with when they are walking (Karasik et al., 2014). However, since this difference was observed through experimenter-recorded video in the home, it is unknown how it generalizes across the day or whether such a difference persists when the experimenter and video camera are absent. Simultaneously recording speech with an audio recorder synchronized with body classification from motion sensors would provide full-day, unobtrusively-collected data to bear on this question.

## 2. Laboratory Study: Validating the Body Position Classification Method

The goal of the laboratory study was to test whether mutually-exclusive body position categories suitable for full-day testing—held by caregivers, supine, prone, sitting, and upright—could be accurately classified from infant-worn inertial sensors. We collected synchronized video and inertial sensor data while infants were in different body positions, and used those data to train classifiers and then validate them against the gold standard (human coding from video observation). As in past work (Nam and Park, 2013; Yao et al., 2019; Airaksinen et al., 2020), our aim was to determine whether the overall accuracy of classification was high (>90% of agreement between model predictions and ground truth data). Moreover, we assessed whether the method could accurately detect individual differences in how much time infants spend in different body positions, which is relevant for characterizing everyday motor experiences and their potential downstream effects on other areas of development (e.g., Soska et al., 2010; Oudgenoeg-Paz et al., 2012; Walle and Campos, 2014).

In order to identify the most accurate method for classifying body position, we compared two modeling techniques: *individual models* that were trained on each individual's data vs. *group models* that used a single model trained on all but one of the participants. Group models are more commonly used in activity recognition studies (e.g., Nam and Park, 2013; Yao et al., 2019; Airaksinen et al., 2020), and have several practical benefits, such as reducing complexity (only needing to train/tune a single model) and providing a generalizable method (group models can be used to classify data in participants for whom no ground truth training data were collected). We reasoned that although individual models take more work to create, they might lead to better accuracy in our use case for several reasons. First, individual models eliminated the possibility that variability in sensor placement across infants could add noise to the data. Second, given the wide range of ages (6–18 months), it allowed us to tailor models to the motor abilities of each infant. For example, the upright category could be dropped for the youngest infants who were never standing or walking. Moreover, the biomechanics of sitting likely differ between a 6-month-old and an 18-month-old, which could result in different motion features. Third, training and validating a model for each infant allows researchers to individually verify the data quality for each infant included in the analyses.

### 2.1. Materials and Methods

#### 2.1.1. Participants

Participants were recruited from social media advertisements and local community recruitment events. The final sample consisted of 15 infants between 6 and 18 months of age (7 male, 8 female, *M* age = 11.28 months). Caregivers reported the ethnicity of infants as Hispanic/Latinx (9) or not Hispanic/Latinx (6). Caregivers reported the race of infants as White (10), More than One Race (2), Asian (1), and Other (1); one caregiver chose not to answer. An additional 7 infants were run in the study but could not be analyzed because of problems with the sensors (one or more sensors failed to record or stream data). Two additional infants were run in the study but excluded due to video recording failures, and one additional infant started the study but did not complete the session due to fussiness. Caregivers were compensated $10 and given a children's book for their infant. The study was reviewed and approved by Institutional Review Board of the University California, Riverside. Caregivers provided their written informed consent to participate in this study and gave permission to record video and audio for both themselves and their infant before the study began.

#### 2.1.2. Materials

Three MetaMotionR (Mbientlab) inertial movement units (IMUs) were placed at the right hip, thigh, and ankle of infants and recorded accelerometer and gyroscope data at 50 Hz. Due to the high rate of sensor failures resulting in participant exclusion, we do not recommend use of this sensor and chose a different sensor for our subsequent projects. The IMU worn on the hip sat inside a clip fastened at the top of the infant's pant leg or diaper on the right side. The other two IMUs were placed in the pockets of Velcro bands strapped to the infant's right thigh (just above the knee) and right ankle. During the study, the IMUs streamed data via Bluetooth to a Raspberry Pi computer running Metabase software (Mbientlab). A camcorder (Sony HDRCX330) held by an experimenter recorded infants' movements throughout the study so that body position could be coded later from video.

#### 2.1.3. Procedure

The study started with synchronizing the three IMUs to the video. To create an identifiable synchronization event in the motion tracking data, an experimenter raised all three sensors together and struck them against a surface in view of the camcorder with both the camcorder and sensors recording. After the synchronization event, the experimenter attached the three IMUs to the infant. The experimenter ensured the correct orientation of the IMUs by checking the arrow indicator on each IMU which faced forward toward the anterior plane with respect to the infant's body position.

After placing the IMUs on the infant, the experimenter guided the caregiver to put the infant in the following positions (assisted or non-assisted): standing upright, walking, crawling, sitting on the floor, lying supine, lying prone, held by a stationary caregiver, held by caregiver walking in place, and sitting restrained in a highchair. Each position lasted 1 min, and the total guided activities lasted approximately 10 min. After the guided activities, the caregivers were asked to play with their infants freely with toys for 5 min. During the free play portion, infants were permitted to move however they wished so that we could record spontaneous body positions. For some infants, the free play portion preceded the guided activities if the infant was fussy or resistant to the guided activities. An assistant held the camcorder and followed the infants throughout the guided and free-play activities to make sure the infant's body was always in view. To check synchronization, a second synchronization event was captured at the end of the study before turning off the video and IMU recordings.

#### 2.1.4. Human Coding of Body Position

**Figure 1:**
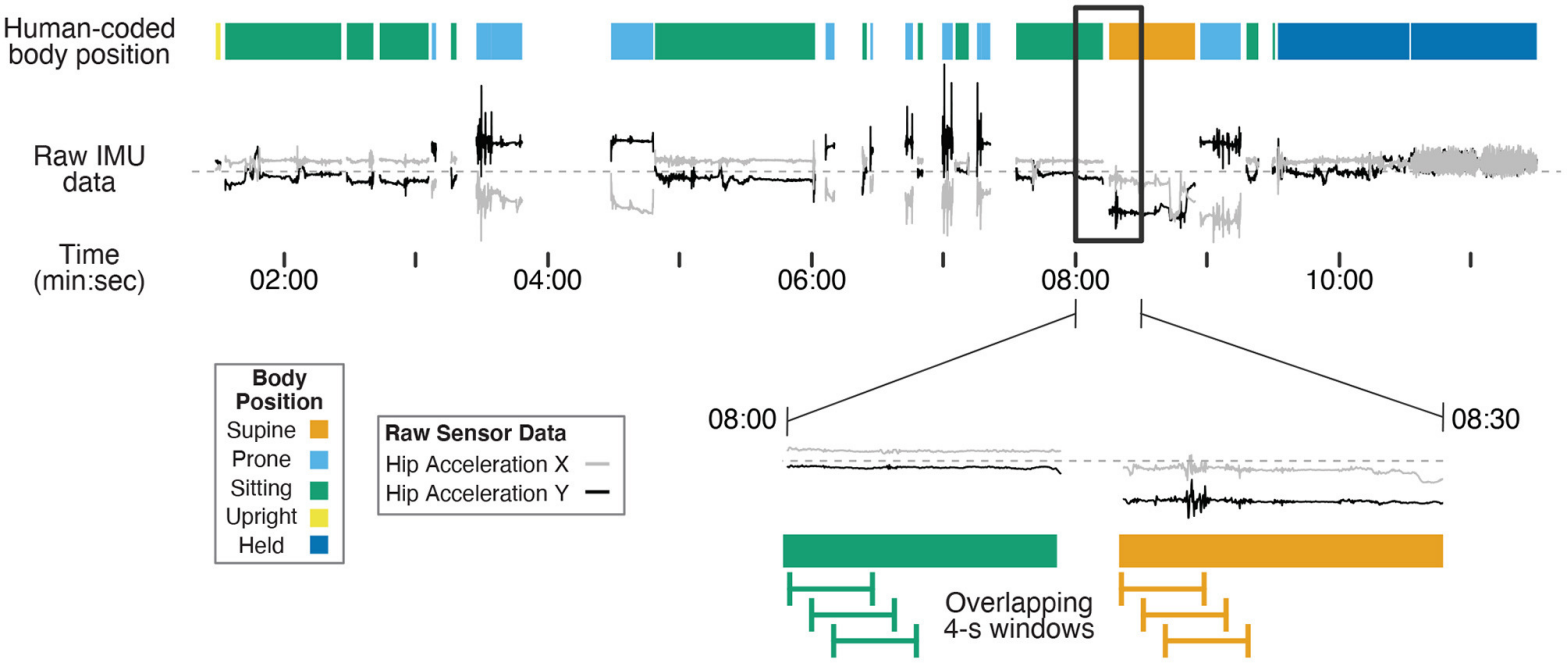
Example timeline showing human-coded body position (colored bars at the top) synchronized with example IMU data (gray and black lines) for one 12-month-old (non-walking) participant's entire session. Two example IMU signals were selected (acceleration in the X and Y axes from the hip sensor) to demonstrate differences in motion data over time in different body positions. The black rectangle shows a 30-s subset of data that are magnified in the bottom timeline. The green and orange lines illustrate 4-s long windows that are shifted in 1-s steps throughout the session to capture discrete body position events. Motion features were calculated within each 4-s window from the raw data to characterize movement in each window for training and prediction. The activities before 7:30 were from the free play portion, and the activities following 7:30 were from the guided portion of the study.

Human coders went through the third-person view videos recorded by the camcorder and identified infants' position in each frame using Datavyu software (www.datavyu.org). Body positions were identified as *supine, prone, sitting, upright*, or *held by caregiver*. Figure 1 shows an example timeline of position codes over the session for one infant.

Supine was coded when the infant was lying on their back. Prone was coded when the infant was lying flat on the stomach or in a crawling position (either stationary or locomoting). Sitting was coded when the infant was sitting on a surface (e.g., a couch or floor, with or without support from the caregiver), the highchair, or on the caregiver's lap. Upright was coded when infants were standing, walking, or cruising along furniture. Held by caregiver was coded when the infant was carried in the caregivers' arms off the ground, excluding times that they were seated on the caregiver's lap. Positions that could not be identified as any of these categories (such as times in transition between body positions) or times where the sensors were briefly removed/adjusted were excluded from coding (i.e., gaps between data in Figure 1). Each video was coded in its entirety by two coders. The interrater reliability between the two coders was high across the 15 videos (overall agreement = 97.6%, *kappa* = 0.966).

#### 2.1.5. Machine-Learning Classification of Body Position

The data were processed in three steps. First, the timeseries of accelerometer and gyroscope data were synchronized to the human-coded body position events. Second, we applied a moving window to the synchronized timeseries to create 4-s long events, and extracted motion features that characterized each event. Finally, we trained random forest classifiers (both individual models and group models) to predict the body position categories for each participant based on the motion features in the 4-s windows.

##### 2.1.5.1. Synchronization

A researcher plotted the accelerometer time series in Matlab and identified the timestamp that corresponded to the acceleration peak at the moment the sensors were struck during the synchronization event. That timestamp was subtracted from the other timestamps to define the synchronization event as time 0. Likewise, Datavyu video coding software was used to find the moment the sensors were struck against the surface in the video, and that time was defined as time 0 for body position codes. In doing so, human-coded body position was synchronized with the motion data. The synchronization event at the end of the session was used to confirm that the synchronization was correct and that no drift correction was needed. The onsets and offsets of each human-coded body position were used to construct a 50 Hz time series of body position categories, providing a body position code that corresponded to each sample of motion data.

##### 2.1.5.2. Window Creation and Feature Generation

As in previous studies in human activity recognition (Preece et al., 2009; Nam and Park, 2013; Airaksinen et al., 2020), overlapping moving windows were applied to the synchronized motion and body position timeseries in Matlab: 4-s windows were extracted every 1 s from the first synchronization point to the end of the session. The magnified timeline at the bottom of Figure 1 shows examples of the overlapping 4-s windows. As such, each 4-s window contained 200 samples of 50 Hz motion data. We omitted any window during which a position category was present for less than 3 s of the 4-s window to avoid analyzing windows that included transition movements between positions or a mix of two different body positions.

Across the 200 samples in a window, we calculated 10 summary statistics—the mean, standard deviation, skew, kurtosis, minimum, median, maximum, 25th percentile, 75th percentile, and sum—for each combination of 3 sensor locations (ankle, thigh, and hip), 2 sensor signals (acceleration, gyroscope), and 3 axes (X, Y, Z for acceleration; roll, pitch, yaw for gyroscope). For example, 10 summary statistics described the ankle's acceleration in the Z dimension. In total, 10 statistics × 3 sensor locations × 2 sensor signals × 3 axes resulted in 180 features. In addition, we calculated the sum and magnitude of movement in each axis across the three sensor locations and the sum and magnitude of movement across axes within each sensor. Finally, we calculated correlations and difference scores between each pair of axes within a sensor and between each pair of sensors for a given axis. These cross-sensor and cross-axis features brought the motion feature total to 204.

##### 2.1.5.3. Model Training

To train and validate *individual models*, each participant's data were separated into a training set that was used to train the model, and a testing set that was held out for validation. In order to mimic the intended use of this method—using video coded at the start of the day to train a model for predicting body position over the rest of the day, we used the first 60% of each participant's data as the training set and the remaining 40% as the testing set. However, because of the sequential nature of our guided activities, selecting the first 60% chronologically would include some activities and exclude others. Thus, we selected the first 60% of data *within each body position category* for the training set to ensure that there were sufficient data to train the models on all positions. To train and validate *group models*, we used a leave-one-out cross-validation technique. A group model was trained using all of the data from 14/15 participants, and then the remaining participant's data served as the testing set. In this way, we could report classification accuracy for each participant (as predicted from a model trained on all other participants). As in Airaksinen et al. (2020) we excluded windows in which the primary and reliability coders disagreed to ensure that only unambiguous events were used in training across both types of models.

Machine learning models were trained in R using the *randomForest* package to create random forest classifiers (Liaw and Wiener, 2002). The random forest algorithm (Breiman, 2001) uses an ensemble of many decision trees—each trained on a random subset of motion features and a random subset of the training data—to avoid overfitting and improve generalization to new cases (Strobl et al., 2009). Prior work shows random forests are well-suited to classifying motor activity (Trost et al., 2018; Yao et al., 2019). By training hundreds of trees on different subsets of features, the classifier detects which features (of our set of 204) are most useful in classifying the categories we chose. In a preliminary step, we optimized two parameters, the number of trees and the “mtry” parameter, by training and testing classification accuracy across a range of parameter values. The optimal number of trees trained in the model was 750 (using more trees took longer processing time without significant gains in model accuracy). The “mtry” parameter refers to how many features are randomly selected in each tree, and the default value was optimal (square root of total number of features). Regardless, performance varied little depending on the values of these parameters. Using the optimal parameters, a random forest model was created based on each participant's training data (individual model) and from all but one participants' data (group model). The *predict* function was then used to apply the model to the motion features in the testing data set to classify each window, and provide a set of predicted categories to compare to the human-coded categories. For individual models, the testing set was the 40% of data held for testing; for group models, the testing set was the “left out” participant. In both cases, testing data were independent from data used to train the model that was validated and included behavior from both the guided activities and the free play portion.

### 2.2. Results

To validate models, we compared the classifier prediction to the ground truth (human-coded body position categories) for each window in the testing data set. The overall accuracy (across body position categories) for each participant was calculated as the percentage of windows in which the model prediction matched the human-coded position. Because windows were of equal length (4 s), accuracy can likewise be interpreted as the percentage of time that was correctly predicted by the model. Table 1 shows the accuracy for each participant for the individual and the group models. For individual models, overall accuracy averaged *M* = 97.9% (*SD* = 2.37%, ranging from a minimum of 92.4% to a maximum of 100%), similar to or exceeding the accuracy reported in related investigations (Nam and Park, 2013; Yao et al., 2019; Airaksinen et al., 2020). For group models, overall accuracy was lower (*M* = 93.2%, *SD* = 0.053), but still strong. A paired samples *t*-test confirmed that individual models yielded superior accuracy, *t*(14) = –3.28, *p* = 0.0055.

**Table 1:** Unweighted, overall accuracy, and Cohen's Kappa for each individual participant in the lab validation study.

**Individual**		**Group**	
**Accuracy**	**Kappa**	**Accuracy**	**Kappa**
0.92	0.91	0.95	0.94
0.94	0.90	0.95	0.91
0.96	0.94	0.84	0.56
0.96	0.96	0.82	0.82
0.97	0.70	0.94	0.81
0.97	0.94	0.99	0.79
0.98	0.94	0.90	0.60
0.99	0.97	1.00	1.00
0.99	0.98	0.98	0.95
0.99	0.99	0.99	0.98
1.00	1.00	0.89	0.84
1.00	1.00	0.91	0.75
1.00	1.00	0.95	0.73
1.00	1.00	0.92	0.88
1.00	1.00	0.94	0.78
0.98	0.95	0.93	0.82

*Accuracy is reported separately for individual vs. grouped models. Bottom row shows average overall accuracy and Kappa values across participants*.

**Figure 2:**
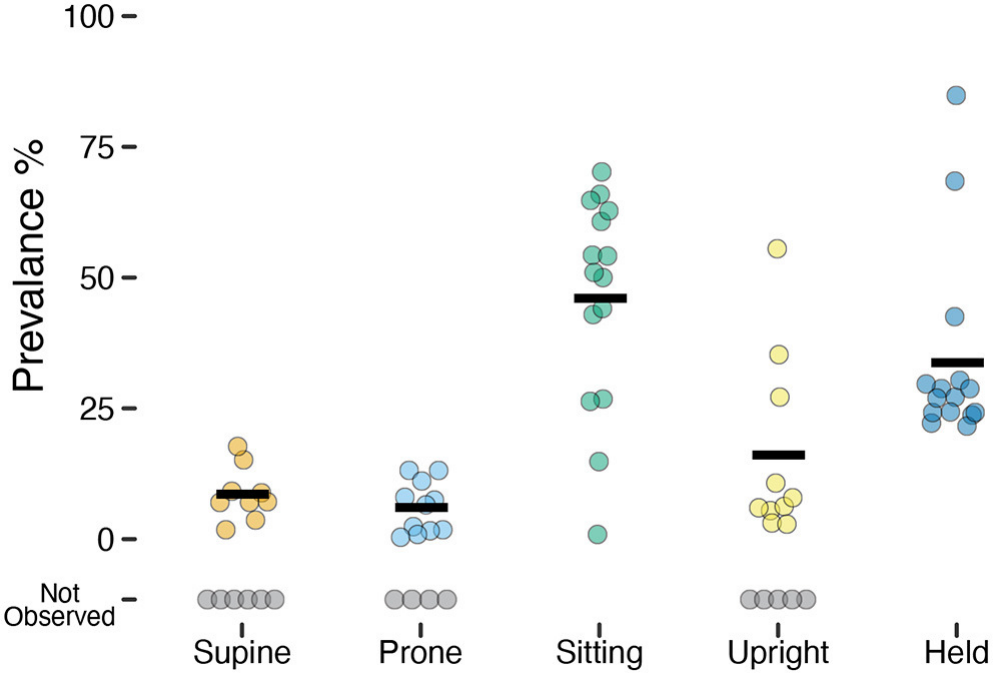
Prevalence of each position observed in the laboratory study. Each individual circle is the prevalence (% of time) for one participant; gray circles indicate participants for whom a position was not observed. Horizontal black lines show the mean prevalence for each position among infants for whom that position was observed.

Although the overall accuracy was excellent, it can overestimate the performance of the model if it does better at predicting more prevalent categories (e.g., sitting) and misses less prevalent categories (e.g., prone). Despite attempting to elicit each body position for a set amount of time for each infant during the guided session, not all infants exhibited each behavior (e.g., infants who could roll might refuse to remain supine and/or prone). Every infant sat and every infant was held by a caregiver, but the prevalence varied greatly across infants of different ages and motor abilities. Figure 2 and Table 2 show the mean prevalence (% of session spent in each position). Infants spent the most time sitting (*M* = 45.98%, 0.9–70.2%) followed by held (*M* = 33.75%, 21.6–84.8%). Upright positions were recorded in 10/15 infants with an average of *M* = 16.02% (out of infants who were upright), and ranged from a minimum of 2.8% to a maximum of 55.5% of the session. Supine (9/15 infants) and prone (11/15 infants) were observed least often. Infants were supine *M* = 8.61% of the time (1.8–17.7%) and were prone *M* = 6.04% of the time (0.4–13.1%).

**Table 2:** Prevalence, sensitivity, and positive predictive value by body position for the lab validation study testing dataset.

		**Sensitivity**				**Positive predictive value**			
**Position**	**Prevalence**	***M***	***SD***	**Min**	**Max**	***M***	***SD***	**Min**	**Max**
Supine	8.61	0.912	0.182	0.500	1.000	1.000	0.000	1.000	1.000
Prone	6.04	0.993	0.022	0.926	1.000	1.000	0.000	1.000	1.000
Sitting	45.98	0.928	0.257	0.000	1.000	0.981	0.036	0.865	1.000
Upright	16.02	0.974	0.043	0.889	1.000	0.967	0.070	0.778	1.000
Held	33.75	0.959	0.064	0.772	1.000	0.976	0.034	0.886	1.000

To account for differences in prevalence, we calculated Cohen's Kappa, a measurement of agreement for classification data that controls for the base rate of different classes. Table 1 shows the Kappa values for each participant, which were significantly higher on average for the individual models (*M* = 0.95, *SD* = 0.076) compared with the group models [*M* = 0.82, *SD* = 0.129, *t*(14) = –3.36, *p* = 0.0047]. As in past work (Greenspan et al., 2021), we interpreted the Kappa values according to Landis and Koch (1977) ranges: 0.81–1.00 “Almost Perfect,” 0.61–0.80 “Substantial,” 0.41–0.60 “Moderate,” 0.21–0.40 “Fair,” 0–0.20 “Slight to Poor.” Based on those guidelines, 14/15 participants' classifications from the individual models were Almost Perfect and 1/15 was Substantial. In contrast, 9/15 participants' classifications from the group models were Almost Perfect, 4/5 were Substantial, and 2/15 were Moderate. Given the better performance of individual models, across both accuracy metrics (overall accuracy and Kappa), we opted to use individual models (and focus solely on those models for the remaining results).

#### 2.2.1. Sensitivity and Positive Predictive Value by Body Position

To better understand the classification performance within each body position, we calculated the *sensitivity* (the proportion of actual occurrences of each body position that were correctly predicted; also referred to as recall) and the *positive predictive value* (the proportion of predictions for a given category that corresponded to actual occurrences; also referred to as precision). Table 2 summarizes sensitivity and positive prediction value (PPV) by position using the individual models.

**Figure 3:**
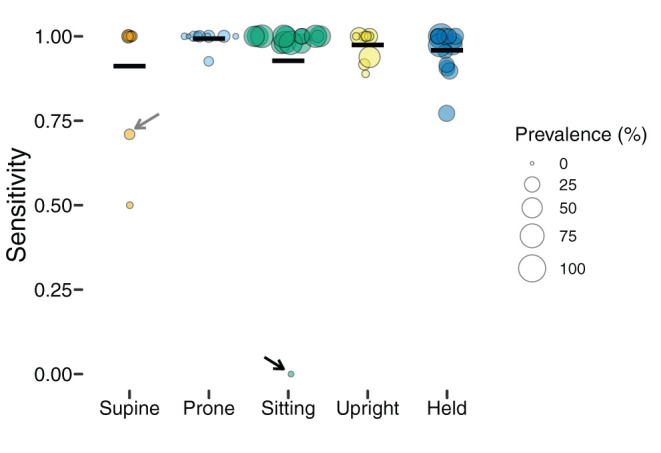
Sensitivity of classification by position in the laboratory study. Each individual circle is the sensitivity for one participant; the size of the point is scaled by the prevalence of that position for that participant (colors indicate body position). Horizontal black lines show the mean sensitivity for each position. Arrows indicate outliers with poor sensitivity that are discussed in text.

Figure 3 shows the sensitivity of classifications by body position, and each individual point shows one participant's data (size is scaled to the prevalence of the position, with larger symbols indicating greater frequency). Although mean sensitivity was generally high (*Ms* > 0.91), there was variability among participants and positions. For example, one infant's supine sensitivity was 0.71 (indicated by the gray arrow), indicating that of the 31 actual supine 4-s windows, the model only predicted 22 supine windows. The worst outlier was one infant's sitting position that had a sensitivity of 0 (indicated by the black arrow). Possibly, sensitivity related to prevalence. For that infant, there were only 2 windows in the testing dataset to classify and both were missed. Because training datasets were similarly limited by the number of windows containing sitting, there were likely insufficient data to train the sitting category for that infant.

**Figure 4:**
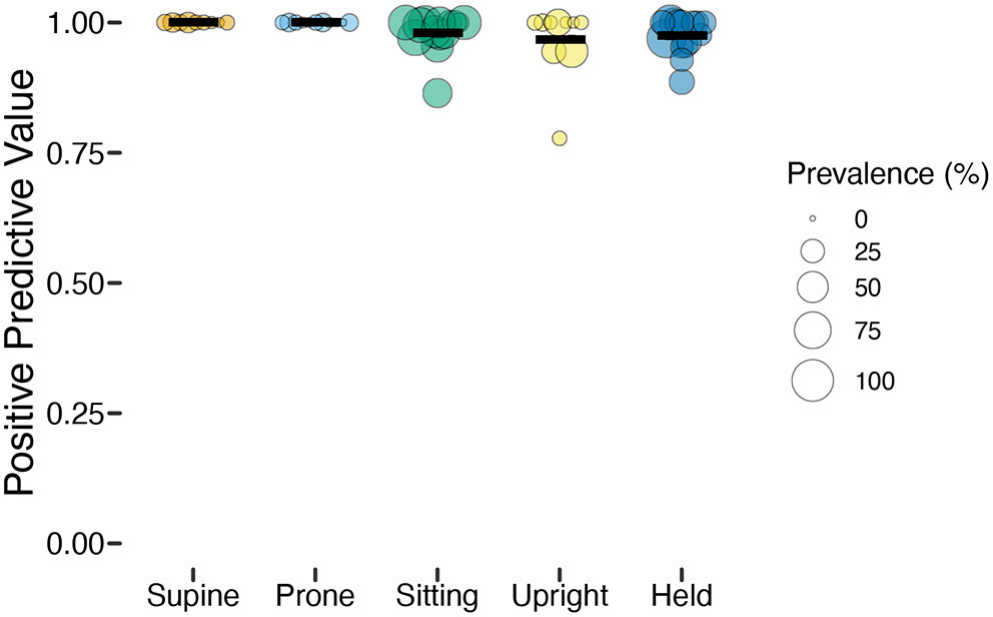
Positive predictive value (PPV) of classification by position in the laboratory study. Each individual circle is the PPV for one participant; the size of the point is scaled by the prevalence of that position for that participant (colors indicate body position). Horizontal black lines show the mean PPV for each position.

Whereas, sensitivity varied among individuals and positions, positive prediction value (PPV) was uniformly high (Table 2). As Figure 4 shows, upright had the worst average PPV (*M* = 0.976) and lowest minimum (0.778). For the participant with the lowest PPV, a value of 0.778 meant that of 9 detected upright windows, only 7 corresponded to actual upright behavior.

Overall, the high (>0.90) average sensitivity and PPV within each class indicate that the classifiers performed well for each position despite varying prevalence. However, there were a few concerning individual outliers for sensitivity. Although outliers such as these might be addressed in future work by collecting and testing with a larger dataset, it is important to know what impact they might have on the interpretation of the data, and in particular, for revealing individual differences in position durations.

#### 2.2.2. Capturing Individual Differences in Position Duration

**Figure 5:**
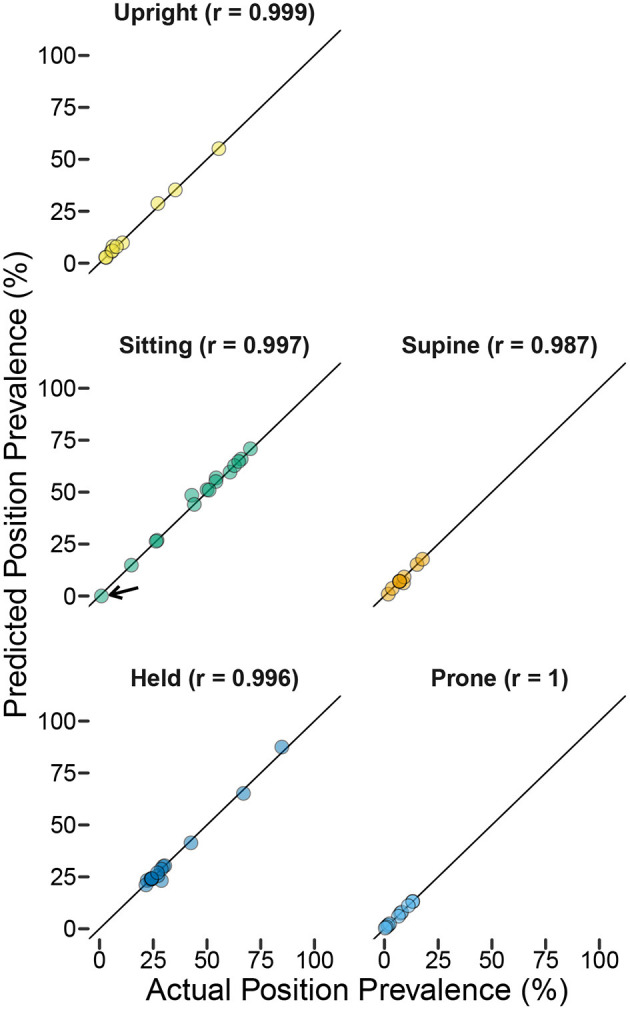
Predicted position prevalence from classification (*y*-axis) plotted against actual position prevalence from human coding of body position (*x*-axis) in the laboratory study. Each graph shows one body position (colors indicate body position), and each symbol represents one participant (titles indicate the *r*-value for the correlation between actual and predicted within each position category). The black arrow in the sitting figure shows the outlier participant with the worst sensitivity from Figure 3.

The intended use of this method is to describe individual differences in the relative amounts of time that infants spend in different body positions. To what extent did the prediction of accumulated time spent in each position reflect the actual time spent in each position? We calculated each participant's predicted prevalence as the proportion of 4-s windows classified in each category divided by the total number of windows in their testing dataset. Figure 5 shows scatterplots of actual vs. predicted prevalence for each position. Correlations (shown in the titles of each scatterplot) were very strong (*r*s > 0.987), indicating excellent consistency between model classification and human coding in detecting individual differences in position prevalence. It is interesting to note that even the most extreme outlier for sensitivity (sitting participant indicated by the black arrow whose sensitivity was 0) did not disrupt the correlation. Since outliers were for participants/positions with low prevalence, missing events (or even missing every event) still resulted in a good-enough predicted value for the purpose of capturing individual differences in posture duration between infants.

## 3. Case Study: Feasibility of Contactless Home Data Collection

The home data collection procedure described below addresses challenges we faced in adapting the laboratory protocol to measuring body position in the home during the COVID-19 pandemic. The risk of COVID-19 transmission between people in an indoor space, especially over prolonged periods of time, meant that the two experimenters could not enter the family's home to place the IMUs, guide the family through the procedures, and control the video camera. Instead, we developed a new, contactless protocol in which the experimenter dropped off equipment outside the family's door and guided the caregiver through procedures over the phone. However, relying on the caregiver to place the IMUs correctly, position the video camera to record infant behavior, and create synchronization events raises additional opportunities for error. Below, we detail several new procedures we developed to address those concerns: designing a customized pair of leggings with embedded IMUs to ensure the sensors are placed correctly by the caregiver, using a 360° camera to capture whole-room video even when camera placement is sub-optimal, and asking caregivers to record daily events that might disrupt IMU recording (i.e., diaper changes and naps).

Although the procedure is similar in many ways to the laboratory study, testing the new method on two case study participants helps to show whether it is feasible to collect high quality data despite major changes to how the procedure was implemented. Major differences between the laboratory study and the home data collection include: using a different set of IMU sensors embedded in a pair of leggings (rather than strapped to the infant), relying on caregivers to correctly place the leggings on the infant, using a fixed camera rather than an experimenter-operated camera to collect training/testing data, asking caregivers to elicit infant body positions and perform synchronization checks in view of the video camera, and collecting data over long periods of time (8 h of home data vs. 15 min of laboratory data). With the experimenter only able to communicate with the caregiver over the phone, any mistakes in equipment placement, synchronization, or body position tasks would not be caught by the experimenter until many hours later when the equipment was retrieved and the experimenter could check the video. As such, we report case study data from two participants to show the feasibility of collecting data (of sufficient quality to build body position classification models) after making these changes. Although we report classification accuracy for those two participants, validation data from a larger sample will be needed to determine if the method consistently allows for accurate body position classification.

### 3.1. Materials and Methods

#### 3.1.1. Participants

Two participants, an 11-month-old infant (Participant A) and a 10.5-month-old infant (Participant B), were tested using the new contactless procedure. Neither infant could walk independently, but both could stand, cruise along furniture, and walk while supported with a push toy or caregivers' assistance.

#### 3.1.2. Materials

To adapt the position classification method for testing in the home during the COVID-19 pandemic, data collection was conducted through a “guided drop-off” procedure. The caregiver received sanitized equipment in a sealed bucket left by the experimenter at their door. The bucket contained 4 Biostamp IMUs (MC10) embedded in a pair of customized infant leggings, a 360° camera on a tripod (Insta360 One R), sanitizing supplies, and paperwork.

The 4 IMUs were placed at the hip and ankle of the infants on both the right and left legs (testing from the lab study revealed that the thigh sensor was the least informative). The Biostamp IMUs are designed for full day recording: They have a long battery life (about 14 h) and record to onboard memory without the need to stream to a device or connect to the internet. Each IMU sensor recorded motion from an accelerometer and gyroscope at 62.5 Hz.

To minimize the possibility of caregivers placing the IMUs incorrectly on infants, a pair of customized leggings were fabricated with 4 small pockets sewn inside the hip and ankle positions of each leg. The snug, elastic fabric kept each sensor tight against the body so that they would not bounce or move independently from the body. The experimenter placed the sensors inside the garment before drop-off to ensure that sensors were oriented and labeled correctly (i.e., sensor A corresponded to the right hip location). The front and back of the garment were clearly labeled so that caregivers would put them on infants in the correct orientation.

We previously relied on an experimenter to operate a handheld camera so that the infant was always in view for body position coding. Without an experimenter in the home, the camera needed to be placed on a tripod. However, that could lead to sub-optimal views and high portions of the time where the infant is out of the video. To address this limitation, we used a camera that recorded in 360° (Insta360 One R). The caregivers were instructed to place the camera on a tabletop tripod in the room where their infants would spend the majority of the day, and were asked to move the camera if the infant left the room for an extended period of time. Since the camera simultaneously records in all directions, the placement of the camera in the room mattered less compared to using a traditional camera with a limited field of view (however, view of the infant could still be obstructed by furniture or people moving around in the room). After the study, the experimenter used specialized camera software to digitally orient the camera so that it exported a video with the infant in view at all times.

The paperwork included the consent form, instructions for how to set up the camera and put on the leggings, and a form that caregivers used to document times when the IMUs were taken off the infants (e.g., diaper changes, naps, excursions out of the home).

#### 3.1.3. Procedure

The procedure consists of a prior-day orientation call, a morning equipment drop-off, an experimenter-guided video session, and a sensor-only recording period for the rest of the day.

##### 3.1.3.1. Prior Day Orientation Call

The participant was contacted a day before participation day to confirm their appointment. During this phone call the experimenter explained the contactless drop-off procedure, gave an overview of the equipment, and explained the consent form to prepare for the participation day.

##### 3.1.3.2. Contactless Equipment Drop-Off

On the participation day, the experimenter brought the equipment bucket—containing sterilized, preconfigured equipment and paperwork—to the participant's home. Importantly, the IMUs were already set to record and were placed correctly within the leggings. When arriving at the participant's door, the experimenter started recording the 360° camera and created a synchronization point by striking the leggings (with the IMUs inside) in view of the camera. Afterwards, the experimenter went back to their vehicle and notified the participant over the phone that the equipment was ready to be picked up.

##### 3.1.3.3. Guided Video Task

While on the phone with the experimenter, the caregiver was asked to open the bucket and then read and sign the consent form. Next, the caregiver was asked to place the 360° camera in an optimal location for video capture (e.g., a coffee table or TV stand). Then, the experimenter asked the caregiver to dress the infant in the leggings and provided prompts to check that the garment was worn correctly.

With all equipment recording, the experimenter (via phone) guided the caregiver through a set of procedures to elicit different body positions for training and testing the classification model. These tasks were the same as the laboratory tasks, but administered by the caregiver instead of the experimenter. The series of guided tasks involved the caregiver placing the infant in different positions: lying on their back (supine), lying on their stomach while stationary (prone), sitting on the floor (with support, if needed), crawling on the floor (if able), walking (if able, caregiver providing support if needed), standing still (if able, caregiver providing support if needed), picking up and holding child off the ground, sitting in a restrained seat (e.g., high chair). Each position lasted approximately 1 min.

Afterwards, the researcher asked the caregiver to create another synchronization event by removing the leggings from their infant, holding the leggings up in the air in view of the camera, and dropping them to the floor. Next, the caregiver was instructed to place the leggings back on their infant and spend 10 min playing with the infant in view of the camera. After receiving those instructions, the phone call with the experimenter ended.

##### 3.1.3.4. Sensor-Only Recording and Material Pick-Up

After the 10 min of free play, the caregiver and infant went about their day as usual with the IMUs continuing to record for the next 8 h or until the experimenter had to pick up the equipment. The only responsibility for the caregivers during the rest of the day was to indicate every time they removed the leggings from the child for any reason (e.g., diaper changes, naps) on the paper log form. This allowed us to omit periods of the day during which the IMUs should not be analyzed.

The 360° camera continued to record until the battery ran out, so the caregiver was asked to position the camera in the room with the infant until the camera stopped recording. The camera could record 90–180 min depending on camera settings we used (in the second case study session we lowered the recording quality to increase recording time). However, because the experimenter started the camera recording before dropping off the equipment on the doorstep, the portion with the infant in view of the camera could vary substantially. For Participant A, the recording lasted 90 min with approximately 45 min of footage of the infant (there was a delay between dropping off the equipment and the camera recording the infant, and the infant went out of view toward the end of recording). For Participant B, we adjusted the settings to record a longer video (the recording lasted 180 min), and the infant was in view for almost the entire 180-min period.

Caregivers could call the experimenter during the day if they encountered any problems. The experimenter scheduled a time to pick up the equipment bucket from the participant's door in the evening or the following morning. All materials were then sterilized following CDC protocols in preparation for the next participant.

#### 3.1.4. Video Processing and Coding

To prepare video data to be coded in Datavyu, an experimenter needed to manually edit the video footage to create a regular field of view video from the 360° video, which was in a proprietary format consisting of two hemispherical video files. Insta360 Studio software allowed the research to select a portion of the 360° video to bring into view. Camera orientations could be tagged at specific times, essentially allowing the researcher to pan the video camera—after the fact—to maintain the infant in view. After exporting a regular field of view video with the infant in view, the coders then identified the infant's position in each frame using the same coding categories as before: supine, prone, sitting, upright, or held by caregiver.

### 3.2. Case Study Results

Each participant's video was coded and synchronized with data from the 4 IMUs worn in the leggings. Data from the guided session (15 min of elicited body positions plus 10 min of free play) were combined and then divided into training and testing datasets. As before, individual models were created using the first 60% of each position type for training the model and the remaining 40% for testing. We compared the predicted positions from the random forest model to the actual coded positions in the testing data to assess the performance of the classifier. The overall accuracy was 85.2% for Participant A (Kappa = 0.80) and 86.6% for Participant B (Kappa = 0.76). Table 3 shows the prevalence, sensitivity, and PPV for each of the five body positions for each participant. Overall, accuracy, Kappas, and sensitivity were weaker compared to the laboratory study, but still within acceptable levels (e.g., Yao et al., 2019; Greenspan et al., 2021).

**Table 3:** Prevalence, sensitivity, and positive predictive value (PPV) by body position for the testing datasets used to assess case studies (Participants A and B).

	**Participant A**			**Participant B**		
**Position**	**Prevalence**	**Sensitivity**	**PPV**	**Prevalence**	**Sensitivity**	**PPV**
Supine	6.91	1.000	1.000	22.74	0.973	0.877
Prone	10.22	0.676	0.833	10.14	0.671	0.728
Sitting	53.59	0.951	0.846	44.29	0.881	0.906
Upright	16.71	0.595	0.758	19.00	0.892	0.835
Held	12.57	0.846	0.928	3.83	0.453	0.837

**Figure 6:**
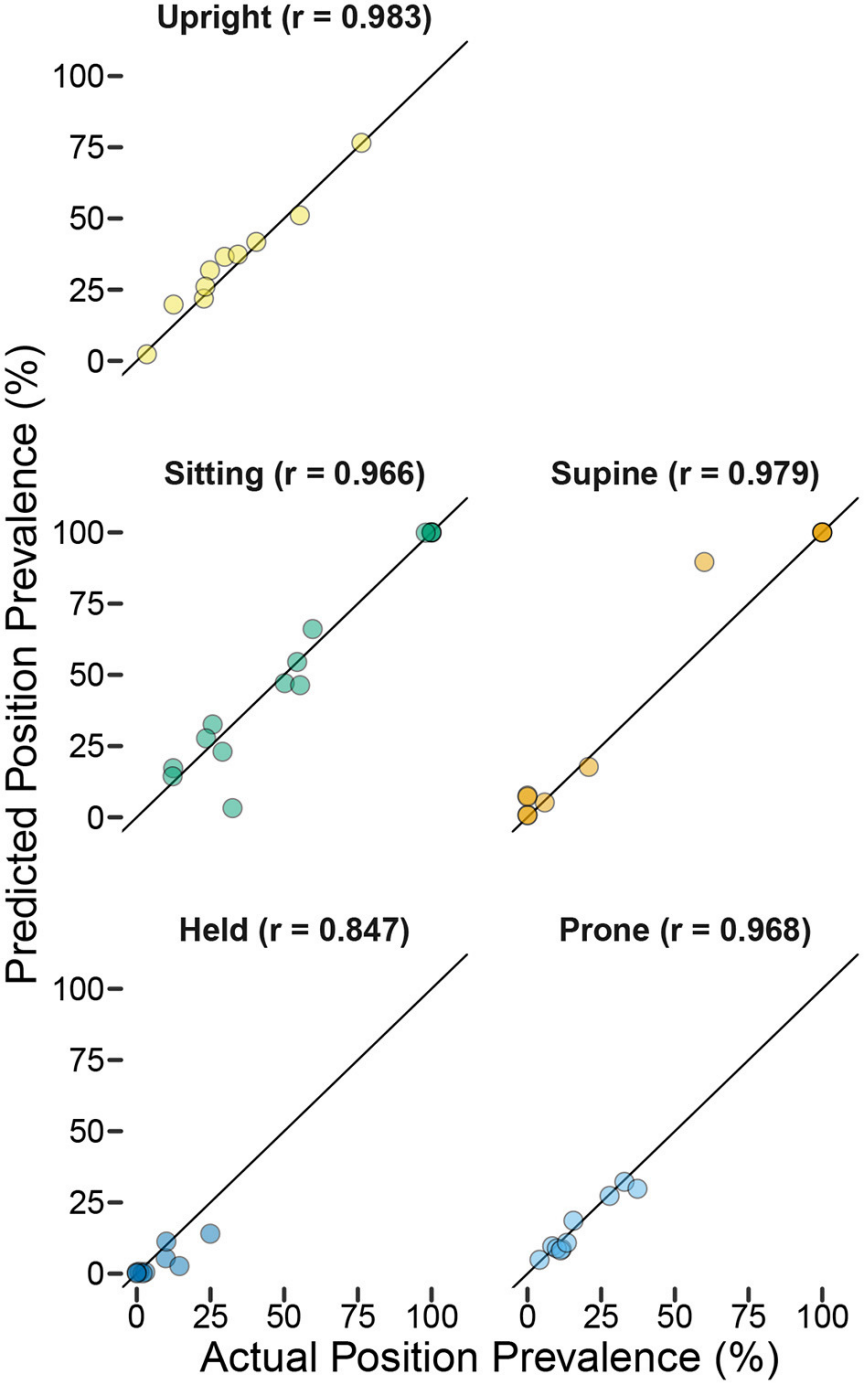
Predicted position prevalence from classification (*y*-axis) plotted against actual position prevalence from human coding of body position (*x*-axis) for home case study Participant B. Each point represents the proportion of time the infant spent in each position during each of 17 7.5-min periods that were video recorded following the end of the training session (titles indicate the *r*-value for the correlation between actual and predicted within each position category). Note that several points are overlapping (e.g., the infant was supine and sitting 100% of the time for multiple periods and was held 0% of the time for multiple periods). The overall correlation between actual and predicted prevalence across positions/periods was *r* = 0.976.

As in the laboratory study, we found that the models performed well at detecting relative differences in the durations of different body positions even when sensitivity was less than ideal. To get a sense of differences in relative durations of positions over time within each infant, we used all available video that followed the guided tasks and free play (e.g., until the battery ran out or the infant was no longer on camera) to code the durations of every body position in 7.5-min intervals. For Participant A, 30 min of video were available (4 7.5-min periods), and for Participant B, 127.5 min of video were available (17 7.5-min periods). Within each period, we calculated the percentage of time in each body position predicted by the model compared to the actual percentage of time coded by hand. Correlations between actual vs. predicted percentages were strong: *r* = 0.911 across positions for Participant A and *r* = 0.976 for Participant B. Within-position scatterplots and correlations are shown in Figure 6 for Participant B, for whom sufficient data were available. Although the correlations were weaker compared to the laboratory study, they suggest that these models can distinguish changes in the relative duration of different positions throughout the day.

**Figure 7:**
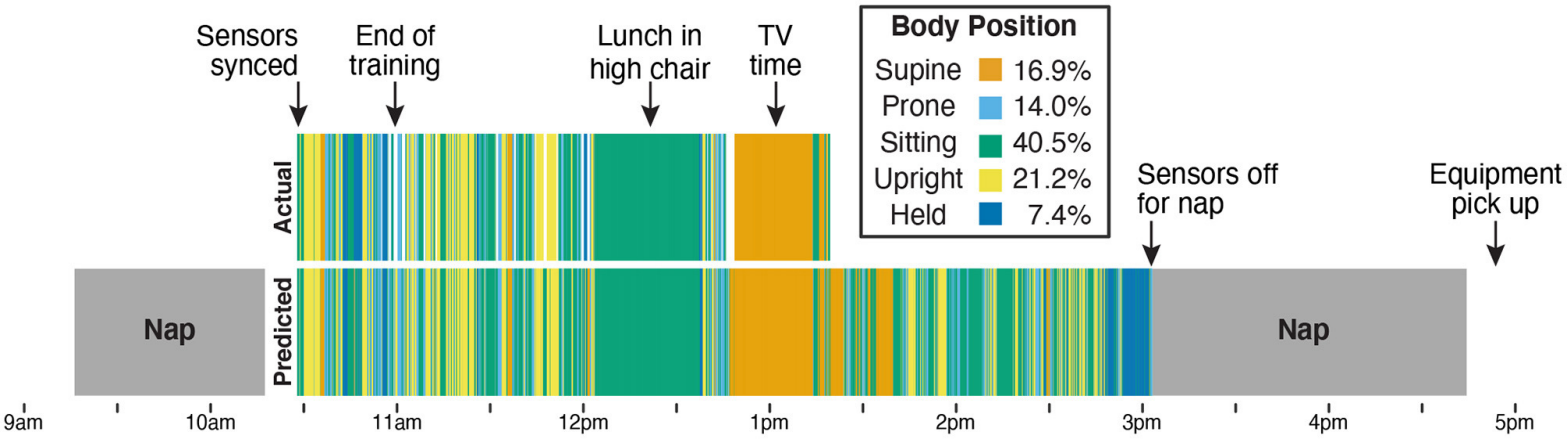
Timeline from Participant B's entire data collection showing actual position codes (top row) compared with model predictions of body position (bottom row). The legend indicates the bar color for each body position and lists the model's prediction of how much time the infant spent in each position over the 4.5-h session. The sensors were placed on the infant after the first nap (at 10:30 a.m.). From 10:30 a.m. to 11:00 a.m., the infant and caregiver were guided through the scripted activities over the phone by the experimenter and completed the prescribed free play in front of the camera. Data from those 30 min were used to train the machine learning classifier. The remaining period (11 a.m. until the camera stopped at 1:15 p.m.) was used for validation. The video recording allowed us to verify that the 40-min period of sitting (from approximately 12 p.m. to 12:40 p.m.) corresponded to a meal with the infant sitting in a high chair and that the period of supine (from approximately 12:45 p.m. to 1:15 p.m.) corresponded to a period of TV viewing while the infant reclined in a seating device. The infant continued to wear the sensors until a nap at 3 p.m., which was the last recorded time before the experimenter picked up the equipment at 5 p.m.

Figure 7 shows a timeline of actual and predicted body positions during the entire recording session for Participant B, providing an example of the type of data afforded by this method. The sensors were synchronized and applied to the infant after her morning nap, and from 10:30 a.m. to 11:00 a.m. the infant and caregiver participated in the guided activities and the required free play portion that were used as training data. The next 2 h (until 1:15 p.m. when the camera battery ran out) were recorded on video and used to calculate the correlations in Figure 6 and the validation statistics in Table 3. We were able to use the video to confirm two notable events in the timeline: A long period of sitting while the infant had lunch in a high chair, and a long period of supine while the infant watched TV in a rocking cradle. The sensors continued to record until the infant took a second nap at 3:00 p.m., and were picked up by the experimenter following the nap. The legend in Figure 7 shows the proportion of each body position predicted by the model across the entire sensor recording period.

## 4. Discussion

The current studies demonstrate the validity and feasibility of classifying infant body positions from wearable inertial sensors. Moving beyond past work that classified only holding events (Yao et al., 2019) or body positions that omitted holding and upright as categories (Airaksinen et al., 2020), our laboratory study classified five body positions that be applied full-day behavior in the home, across activities that may include different forms of each body position (e.g., sitting on the floor during play, sitting in a high chair during a meal). Although sensitivity varied among participants and body positions, the classification system was able to reveal individual differences in time spent between different body positions between infants. The case studies went a step further to provide a proof-of-concept of how the method could be employed in the home across a long recording period. For both case study participants, we successfully collected video and motion data in the home by guiding caregivers through a contactless equipment drop-off procedure. The resulting body position classifiers—trained from data in which no experimenter entered the home or operated the equipment—were sufficiently accurate to measure intra-individual changes in body position over time, suggesting that the procedure could be carried out successfully by caregivers who received instructions over the phone.

Full-day recordings of body position have the potential to transform our understanding of everyday motor behavior in a similar way that wearable audio recorders have changed the study of language development. Wearable audio recorders capture the entire day (or even multiple days) of language input in the home (Weisleder and Fernald, 2013). The language input infants receive differs between the lab and real life, depends on the activity context, and can be biased by the presence of an experimenter (Tamis-LeMonda et al., 2017; Bergelson et al., 2019). Moreover, recorders such as the LENA automatically score metrics about language input to reduce the need for laborious transcription. Although our method of body position classification still depends on collecting and scoring video data, a 30-min training period at the start of the day is enough to then turn off the cameras and unobtrusively record and classify body position for the remainder of the day (or in the future, multiple days).

As real-time, full-day motor experience data become available, what might we learn? Although Figure 7 shows “only” 8 h in the life of one infant, it is striking to observe the heterogeneity in motor activities across the day. The late morning and early afternoon were marked with frequent changes between different positions as the infant engaged in unrestrained play. In contrast, the lunch and TV times created long, interrupted bouts of a single body position. As more data become available from infants of different ages, motor abilities, and caregivers, we expect to see large inter- and intra-individual differences in body position. Indeed, our ongoing work using ecological momentary assessment to record infants' activities (e.g., play, feeding, media viewing, errands, etc.) shows that play is more frequent than any other activity for 11- to 13-month-olds (feeding is the second most prevalent), but play time differs greatly between infants (Kadooka et al., 2021). Some infants played for one third of the waking day, whereas others played for two thirds. Most likely, differences in daily activities provide a partial explanation for why body position rates measured in laboratory play (Thurman and Corbetta, 2017; Franchak et al., 2018) do not correspond to those measured in full-day EMA surveys (Franchak, 2019). Full-day timelines from wearable sensors will be even better suited to explain differences between the laboratory and the home because they provide dense, real-time data (tens of thousands of samples a day) compared with the 8–10 total samples yielded through EMA notifications every hour.

Although the results of our validation and case studies are promising, there is still reason to be cautious as we apply the method to full-day testing in the home. In both the laboratory and home case studies, sensitivity was poor for few positions for a few participants. Although it was encouraging that those cases did not preclude us from observing inter- and intra-individual differences, more testing—particularly in a larger set of home participants—will be needed to know how robustly our method can deal with poor classifications. Whereas, outliers in many measures used to assess individual differences must simply be trimmed based on a distributional assumption (e.g., extreme CDI scores, Walle and Campos, 2014), our method relies on collecting ground truth data for every individual. Since each individual infant's model can be validated, we have a principled way of excluding outliers based on the prediction accuracy for each infant, each body position, and each session. But, training individual models comes with a cost: It relies on collecting video data for every participant, training those videos, and fitting individual models. It is possible that when a larger set of training data are available, that the accuracy of group models will approach that of individual models. Or, sub-group models could be made to make predictions in infants of the same age (or who share the same repertoire of motor behaviors). Unfortunately, insufficient data were collected from infants of different age groups to test a sub-group approach.

Regardless, future work should investigate why those fits were poor with an eye toward reducing erroneous predictions (instead of excluding data *post hoc*). One possibility is that not enough data were available to train the model for those positions. Although we attempted to elicit different body positions in every infant, infants were not always cooperative. For example, infants who can crawl and walk may be unhappy lying on their backs for minutes at a time. As the time of recording becomes longer, it also creates greater opportunity for errors to arise (such as a caregiver putting on the leggings the wrong way after a diaper change or nap). We hope that by asking caregivers to document such events, we will be able to exclude portions of the day with erroneous data. In the future, collecting validation data (with video) intermittently through the day or at the end of a session could provide a more objective way to check the robustness of the classifier. Given the complexity of testing behavior in the wild, decrements in accuracy for the case study participants (from 98% in the laboratory to 85% in the home) were to be expected. Although it is encouraging that accuracy was still at an acceptable level in the case study participants, more data will be needed to demonstrate whether the method is accurate across a larger sample of participants in the home. Individual differences in infants' motor repertoires and daily routines/activities likely add to heterogeneity in body position frequency, and whether such variability can be captured across a large sample in the home remains to be tested.

Generalizing from training data—a portion of which contained elicited positions—to unconstrained, free-flowing behavior is a significant challenge. As noted, it is especially difficult when sufficient data for all categories to train and test the models are not available for every infant. One strategy that we used to deal with the unpredictable nature of infant data collection was to design a two-part training procedure—a guided task that attempted to gather data from a fixed list of behaviors followed by a free-play procedure that gathered data from infants in more free-flowing, self-selected positions. Ideally, this two-pronged approach would provide complementary data: In the guided section, the caregiver would place infants in positions that would be rare in free play, such as holding infants and restraining them in a high chair, and free play would capture more naturalistic behavior. However, a limitation of this approach is that we trained and tested models using both guided and free play data. A stronger test would have been to assess model performance on a set of completely naturalistic data (such as a period of free play or home life that excluded any elicited behaviors). Because our approach relied on training models using both types of data, we could not do this in our dataset—there was not enough free play data collected to hold it in reserve for testing. In future work, collecting a separate set of naturalistic testing data would provide a more stringent test of how well models will generalize to body position in daily life.

In addition to providing proof-of-concept data, our two home case studies also highlight the utility of a contactless equipment drop-off procedure for studying infant home behavior. Many infant development researchers—especially those who use looking time metrics—can turn to video conferencing or toolboxes such as Lookit (Scott and Schulz, 2017) for a substitute for in-person studies. In contrast, for researchers who study gross motor behaviors, such as walking and crawling, it may be difficult or impossible to make the paradigm fit on a computer screen. Cameras fixed on a tripod are not ideal for capturing motor behavior, which is why home observation studies typically rely on an experimenter to record infants as they move from place to place (Karasik et al., 2011). Although the 360° cameras we used in the home case studies cannot follow the infant from room to room, they do provide a way to digitally pan and follow the infant. Moreover, the sensors themselves move with infants from place to place, obviating the need for an experimenter to follow infants around. There is no doubt that this method would be easier to implement in person. Although caregivers successfully placed the cameras and leggings on infants, having an experimenter in the home would reduce the burden on the caregiver. In the ideal scenario, the experimenter would briefly visit the home to place the equipment, and then data could be recorded for the rest of the day without the experimenter present.

In summary, characterizing the inputs for development—what infants do and experience on a daily basis—strengthens our ability to build theories (Dahl, 2017; Oakes, 2017; Franchak, 2020). We identified a new way of capturing one type of input, body position, and expect that measuring daily body position experiences will help reveal how infants' burgeoning motor skills are linked with cascading effects on language and spatial cognition (Soska et al., 2010; Oudgenoeg-Paz et al., 2012, 2015; Walle and Campos, 2014; West et al., 2019). In the future, wearable sensors may be used to build machine learning classifiers for other behaviors, such as locomotion (time spent crawling and walking) and manual activities. In combination with other wearable equipment, such as “headcams” and audio recorders, we may better understand how infants shape the multi-modal inputs for learning through their own actions.

## Data Availability Statement

The datasets presented in this study can be found in online repositories. The names of the repository/repositories and accession number(s) can be found below: OSF repository: https://osf.io/wcga9, doi: 10.17605/OSF.IO/WCGA9.

## Ethics Statement

The studies involving human participants were reviewed and approved by Institutional Review Board of the University of California, Riverside. Written informed consent to participate in this study was provided by the participants' legal guardian/next of kin.

## Author Contributions

JF, VS, and CL contributed to the conception and design of the study. VS collected and coded study data. JF completed the statistical analyses. All authors wrote sections of the manuscript, revised, read, and approved the submitted version.

## Funding

This project was funded by National Science Foundation Grant BCS-1941449 to JF.

## Conflict of Interest

The authors declare that the research was conducted in the absence of any commercial or financial relationships that could be construed as a potential conflict of interest.

## Publisher's Note

All claims expressed in this article are solely those of the authors and do not necessarily represent those of their affiliated organizations, or those of the publisher, the editors and the reviewers. Any product that may be evaluated in this article, or claim that may be made by its manufacturer, is not guaranteed or endorsed by the publisher.
